# A putative chordate luciferase from a cosmopolitan tunicate indicates convergent bioluminescence evolution across phyla

**DOI:** 10.1038/s41598-020-73446-w

**Published:** 2020-10-20

**Authors:** Michael Tessler, Jean P. Gaffney, Anderson G. Oliveira, Andrew Guarnaccia, Krista C. Dobi, Nehaben A. Gujarati, Moira Galbraith, Jeremy D. Mirza, John S. Sparks, Vincent A. Pieribone, Robert J. Wood, David F. Gruber

**Affiliations:** 1 Sackler Institute for Comparative Genomics, American Museum of Natural History, New York, NY 10024 USA; 2 Department of Biology, St. Francis College, Brooklyn, NY USA; 3 Department of Natural Sciences, Baruch College, City University of New York, New York, NY 10010 USA; 4 The Graduate Center, PhD Program in Biology, City University of New York, New York, USA; 5 Departamento de Oceanografia Física, Química e, Geológica, Instituto Oceanográfico, Universidade de São Paulo, São Paulo, 05508-120 Brazil; 6 Institute of Ocean Sciences, 9860 West Saanich Road, P.O. Box 6000, Sidney, BC V8L 4B2 Canada; 7 Departamento de Química, Instituto de Ciências Ambientais, Químicas e Farmacêuticas, Universidade Federal de São Paulo, Diadema, São Paulo, Brazil; 8 Division of Vertebrate Zoology, Department of Ichthyology, American Museum of Natural History, New York, NY 10024 USA; 9 Cellular and Molecular Physiology, Yale University, New Haven, CT USA; 10 Wyss Institute for Biologically Inspired Engineering, Harvard University, Cambridge, MA USA

**Keywords:** Biochemistry, Ecology, Evolution, Molecular biology, Ocean sciences, Engineering

## Abstract

Pyrosomes are tunicates in the phylum Chordata, which also contains vertebrates. Their gigantic blooms play important ecological and biogeochemical roles in oceans. *Pyrosoma*, meaning “fire-body”, derives from their brilliant bioluminescence. The biochemistry of this light production is unknown, but has been hypothesized to be bacterial in origin. We found that mixing coelenterazine—a eukaryote-specific luciferin—with *Pyrosoma atlanticum* homogenate produced light. To identify the bioluminescent machinery, we sequenced *P. atlanticum* transcriptomes and found a sequence match to a cnidarian luciferase (RLuc). We expressed this novel luciferase (PyroLuc) and, combined with coelenterazine, it produced light. A similar gene was recently predicted from a bioluminescent brittle star, indicating that RLuc-like luciferases may have evolved convergently from homologous dehalogenases across phyla (Cnidaria, Echinodermata, and Chordata). This report indicates that a widespread gene may be able to functionally converge, resulting in bioluminescence across animal phyla, and describes and characterizes the first putative chordate luciferase.

## Introduction

**Figure 1:**
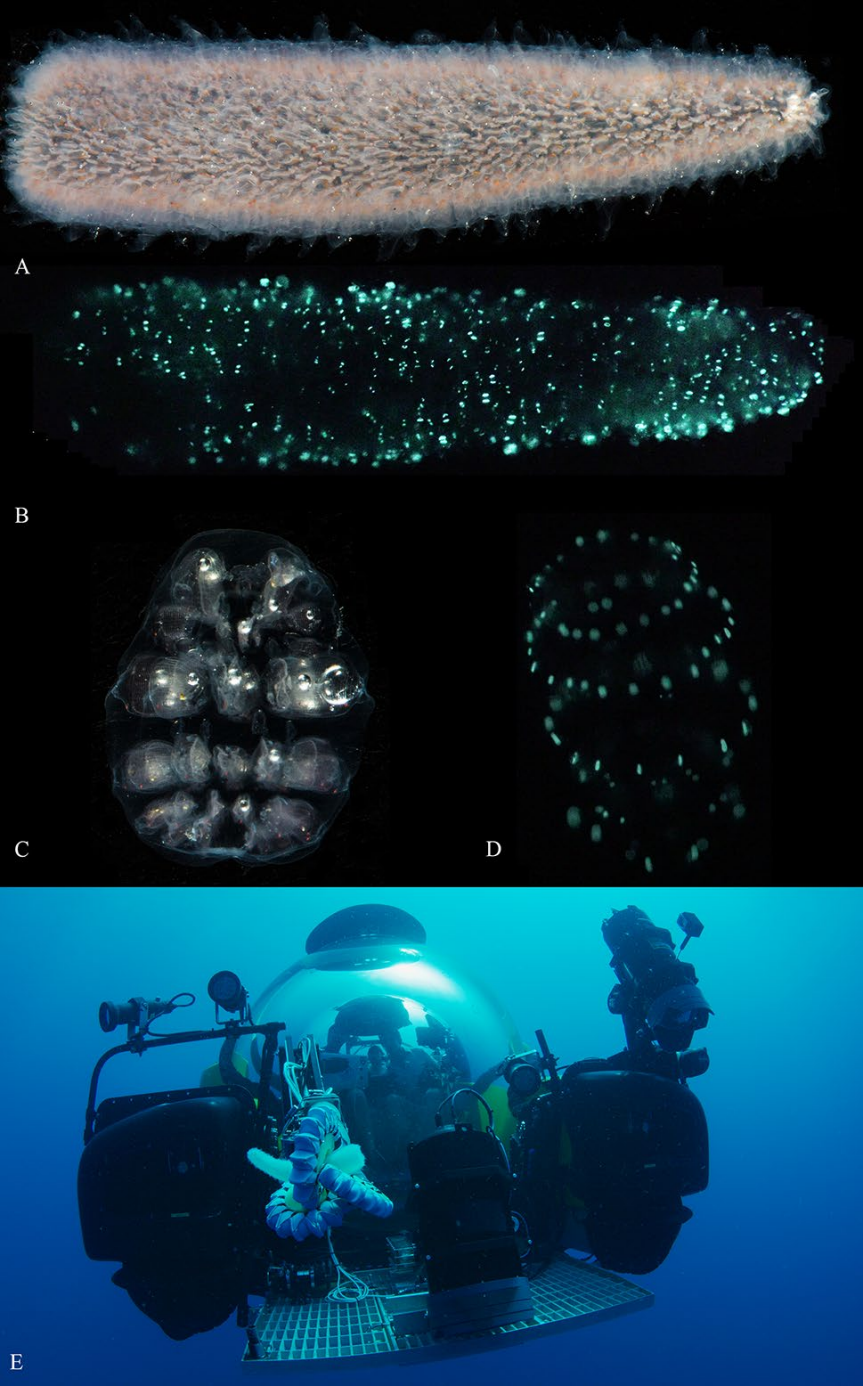
Pyrosomes—*Pyrosoma atlanticum* (**A**,**B**; ~ 155 mm × 40 mm) and *Pyrosomella verticillata* (**C**,**D**; ~ 25 mm × 40 mm)—from SE Brazilian Atlantic under (**A**,**C**) white light and (**B**,**D**) producing bioluminescence following mechanical stimulation; (**E**) soft robotic arm collection of *Pyrosoma atlanticum* from the NE Brazilian Atlantic from *Nadir* (Triton 3300/3 submarine).

Pyrosomes are colonial, pelagic tunicates known for their exceptionally sustained bioluminescence and their sporadic, yet massive blooms^1–3^ (Fig. 1 and Supp. Video 1, 2). The name pyrosome, which in Greek translates as “fire-body”, is derived from their unique bioluminescent displays. This hallmark feature was eloquently described by Thomas Henry Huxley, then a 25-year-old Assistant Surgeon onboard the HMS Rattlesnake, as “miniature pillars of fire gleaming out of the dark sea”^4^. While pyrosomes attracted considerable interest of naturalists in the seventeenth and eighteenth centuries^5–7^, many of the most basic facts about their bioluminescence remain elusive. A current leading hypothesis is that bioluminescence in pyrosomes is derived from bacterial symbionts^8–10^. Understanding the biochemical pathway for pyrosome bioluminescence is of noteworthy interest as it represents a bioluminescent chordate, in the subphylum that is the sister group to vertebrates. The only instances of bioluminescence in vertebrates occur in some elasmobranchs and bony fishes. In this manuscript, our goal is to explore the biochemical mechanism of bioluminescence in a pyrosome (*Pyrosoma atlanticum*) and attempt to place this mechanism in an evolutionary context. To do this, we combined transcriptomics, phylogenetics, immunohistochemistry, gene synthesis of a novel luciferase, and tests of luciferase enzymatic activity. Specimens were obtained in this study at similar times with a soft-robotic-equipped submarine^11^ (Fig. 1E) and Isaacs-Kidd Midwater Trawl off of Brazil and via standard trawl methodologies from a rare bloom in Canada^12,13^ (Supp. Figure 1).

Pyrosomes are in the subphylum Tunicata, which comprises filter-feeding marine chordates (~ 3000 species) and is the sister taxon to our subphylum, Vertebrata^14^. The class Thaliacea consists of ~ 100 species distributed across doliolids, salps, and, our focal organisms, the pyrosomes^14^. *Pyrosoma atlanticum* is one of four currently recognized species in the genus.

The extreme blooms of *P. atlanticum* throughout temperate and tropical waters^1–3^ are thought to play an important role in oceanic carbon cycles; specifically, the numerous individuals are carbon dense for gelatinous organisms and sink rapidly, thus transporting carbon from coastal margins and pelagic zones to benthic zones like the deep sea^2,15^. Pyrosomes have shown the capacity to consume > 50% of phytoplankton in the upper 10 m of the water column^16^ and a single *P. atlanticum* colony can clear 35 L per hour^17^. During blooms, the jelly fall of *P. atlanticum* is then consumed by numerous animal phyla^2,15^. Apart from extensive blooms, pyrosomes can still be a significant food source for at least 62 species of fishes and three species of marine turtles^18^. While it is mainly observed in the upper photic zone, *P. atlanticum* has been reported to depths of 1000 m^1^. In this study, the *P. atlanticum* specimens collected from Brazil were sparsely distributed and individuals were only observed and collected on a few occasions. In comparison, *P. atlanticum* obtained from off of Vancouver Island (Supp. Figure 2) were collected during one of the most extensive blooms in recorded history, coating oceanographic sampling gear, clogging fishing nets, and exceeding over 200,000 kg/km^3^ in biomass^12^.

**Figure 2:**
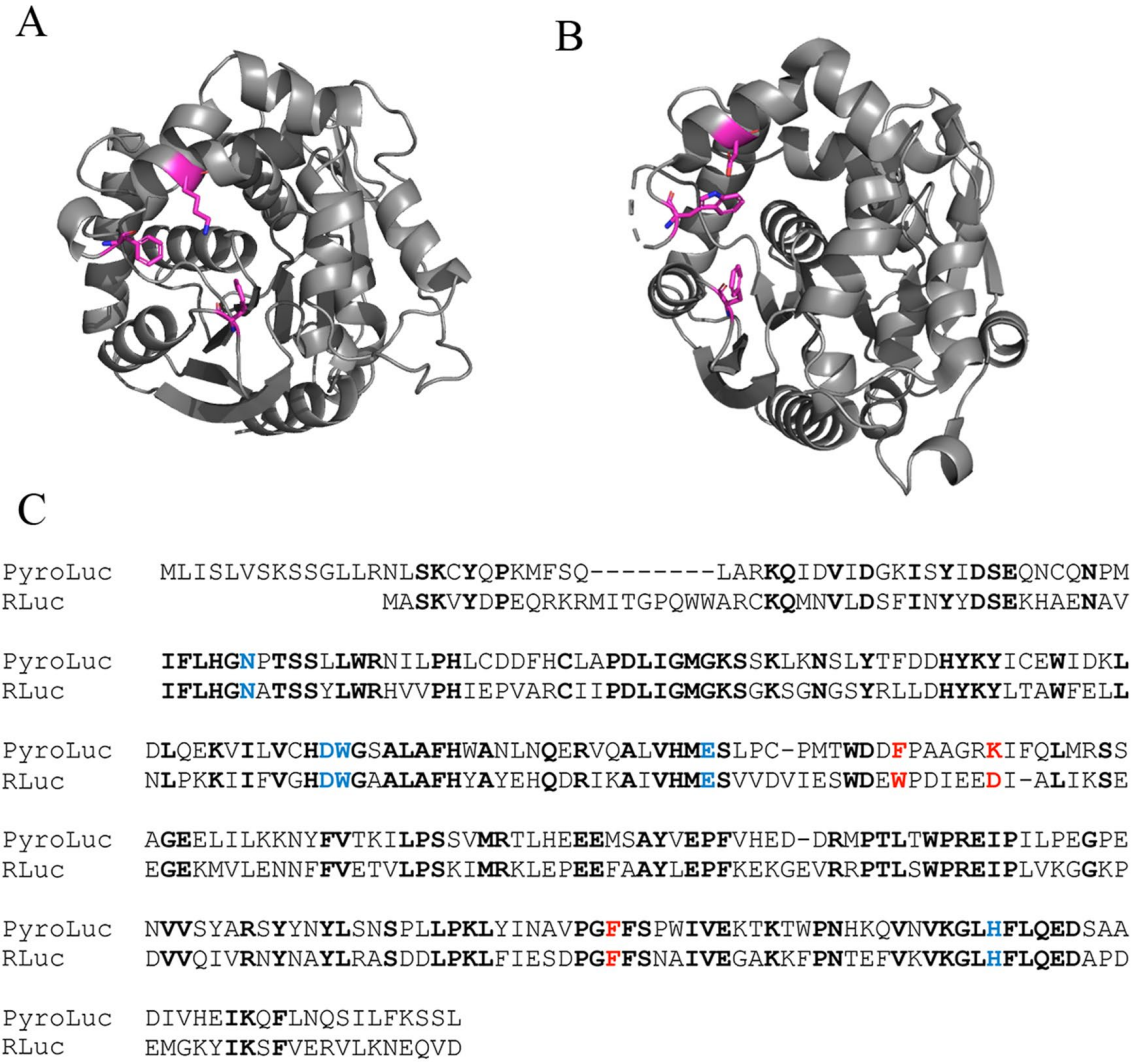
(**A**) Predicted model of PyroLuc created using SWISS-Model based on *Renilla* luciferase and (**B**) model of *Renilla* luciferase (2PSJ^33^). Both models were rendered in PyMOL 2.3 (https://pymol.org). Magenta sticks show conserved active site residues in the coelenterazine binding site. (**C**) Alignment of PyroLuc and RLuc. The residues highlighted in blue make up the catalytic triad in RLuc and those in red represent those in the coelenterazine binding site. Bold type represents identical residues.

Contextualizing our main focus—bioluminescence—pyrosomes stand out as well. Pyrosomes are one of a few organisms known to exhibit bioluminescence in response to light (Supp. Video 3), along with dinoflagellates^19^ and euphausiid shrimp^20^. Pyrosome light can also be triggered by more typical sources, such as electrical, mechanical, and chemical stimuli^21^ (Fig. 1). Each zooid per colony has two regions of light-producing cells on the sides of the intake siphon^10^, making light production tightly linked with colony size^21^. The blue-green light emitted by *P. atlanticum* has been reported to have a peak emission at 475 nm^22^, 485 nm^23^, and 493 nm^24^.

Given their propensity to respond to light, pyrosomes are the only known colonial organisms where bioluminescence is associated with communication between the zooids in a colony^25^. Furthermore, pyrosome colonies have been shown to respond to the bioluminescence of conspecifics^7^. This way of using light for intraspecific communication is well described in non-colonial marine species, such as polychaetes, ostracods, and fishes^26,27^. The serial photic excitation of pyrosome zooids results in a wave of bioluminescence that travels at 2.1–4.1 mm/s across the colony^22^. This phenomenon was first noted in the 1800s^6^ and can be seen in Supp. Video 1 and 2 in *P. atlanticum* and *Pyrosomella verticillata*. When the light flash is absorbed by the eyes of neighbouring zooids, they both emit light and arrest ciliary movement, which ceases propulsion^10^. While it can be presumed that this response enables zooids to close down and stop filtering when exposed to harmful stimuli, this behavior has not been confirmed by observation in a natural setting. One possible explanation as to why such behavior might be beneficial is that pyrosomes could use their light emissions as ‘burglar alarms’; similarly densely populating organisms sometimes appear to use bioluminescence to prompt second order predators to come after their attackers^8^. Given the propensity of pyrosomes to form dense blooms, such a tactic might be aided by nearby colonies producing their own bioluminescence.

Regardless of the function behind pyrosome bioluminescence and the tissue localization, the exact mechanism has not been determined. Like other bioluminescent organisms, pyrosomes rely on a chemical reaction between a substrate (luciferin) and an enzyme (luciferase) to produce their light; however, the specific luciferin and luciferase have yet to be identified^8^. Bacterial-bodies have sometimes been implicated as the causative agent behind pyrosome light emission, but that explanation has been debated since the early 1900s^10,28^. The results we present below advance this debate, suggesting that *P. atlanticum* has an endogenous luciferase that is related to the presumed haloalkane dehalogenases of other invertebrates. The type of endogenous enzyme is also found in both bacteria and eukaryotes, and appears to have evolved into luciferases in two other invertebrate lineages^29^. Their more typical function is to break carbon–halogen bonds^30^.

## Results

### Transcriptomic sequencing and analysis

Assembled transcriptomes (Illumina HiSeq sequences) for Brazilian sample 2B had 152,084 contigs with a total of 75,635 ORFs while sample 2C had 134,746 contigs with a total of 70,340 ORFs; the Canadian sample P2 had 227,360 contigs with a total of 112,334 ORFs while sample P3 had 206,824 contigs with a total of 104,057 ORFs. The large number of ORFs corresponds to the fact that we used a 5 amino acid minimum to allow for searches for other proteins of interest that may be short.

Of the transcriptomes, one from Brazil (2B; identity = 48%; e = 3.8^−46^) and one from Canada (P3; identity = 48%; e = 6.67^−97^) had ORFs that matched the “Chain A, Crystal Structures Of The Luciferase And Green Fluorescent Protein” of the sea pansy, *Renilla reniformis* (PDB accession = 2PSF), also known as RLuc. However, when comparing the 2B sequence to nr in GenBank rather than Swissprot/Uniprot, the sequence was less clearly a luciferase than a haloalkane dehalogenase (48% vs. > 50% identity). The Canadian sequence P3 that matches RLuc is hereafter referred to as PyroLuc; the Brazilian sequence is named PyroB.

For generating homology models, Swiss-Model utilized *Renilla* luciferase accession 2PSJ (Fig. 2). The models were rendered using PyMol 2.3 (pymol.org). This RLuc luciferase is evolutionarily related to the α/β hydrolase family with close homology^31,32^. Sequence alignment of PyroLuc and *Renilla* luciferase (2PSJ^33^) showed conservation of the catalytic triad and the active site (Fig. 2).

The alignment of PyroLuc with 2PSJ shows the secondary structure around the binding pocket of colenteramide to be similar to that of 2PSJ and the colenteramide molecule seems to fit well in the binding pocket (Fig. 2). For PyroB there is a shift in the secondary structure as compared to the 2PSJ, which may cause a shift in the binding pocket of colenteramide. Accordingly, PyroLuc was used for downstream expression, while PyroB was not.

Samples P2 (65% identity and e = 1.66^−50^) and P3 (62% identity and e = 8.03^−89^) also match a luciferase from a *Pleuromamma* sp. (AAG54096), which is known to exhibit bioluminescent properties (Patent: US 6232107-B 15-MAY-2001). While these are rather good quality matches, neither one possessed start codons and were accordingly not used for downstream analysis or expression testing.

### Pyrosome luminescence experiments

**Figure 3:**
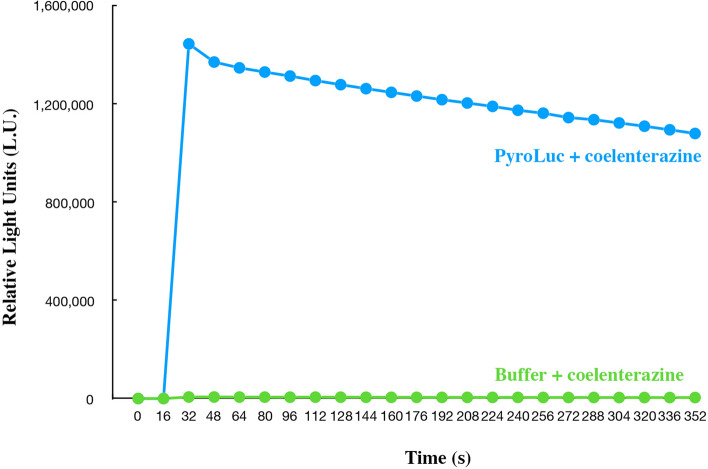
In blue, luminescence reading of purified PyroLuc (3.2 μM) following the addition of coelenterazine (24.5 μM). PyroLuc and coelenterazine were diluted in PBS with 300 mM imidazole, pH 7.4. In green, PBS buffer control with addition of 24.54 μM coelenterazine. Coelenterazine was injected at 16 s for both experiments.

Mixing coelenterazine with *P. atlanticum* homogenate produced a luminescent reaction (Supp. Figure 3). Purified protein was used for luminescence experiments. Figure 3 shows a representative trial of the PyroLuc luminescence: 3.2 µM PyroLuc was used with 24.54 µM of coelenterazine, resulting in a luminescence reading of 1.5 × 10^6^ relative light units (RLU). To confirm enzymatic activity, we conducted several controls. We boiled the purified PyroLuc sample, which resulted in 5.4 × 10^2^ RLU. In addition, we purified a protein, matrix metalloproteinase-7 (MMP7), unrelated to bioluminescence under the same conditions and did not observe significant light emission (Supp. Figure 4). Buffer controls were also performed, using buffers involved in all purification steps. NanoLuc, an optimized luciferase (Promega), was expressed in our lab and was used as a positive control in all experiments. We used matrix metalloproteinase-7 as a control for the luminescence experiments given it has no known luminescent properties. The expressed PyroLuc produces significantly more light than in controls. For the control of matrix metalloproteinase-7, we saw values of ~ 1.5 × 10^4^. For PyroLuc we saw a peak luminescence reading of 1.4 × 10^7^. The concentrations were 4.32 μM MMP7 for elution 1 and 0.57 μM MMP7 for elution 2 in PBS, pH 7.4.

**Figure 4:**
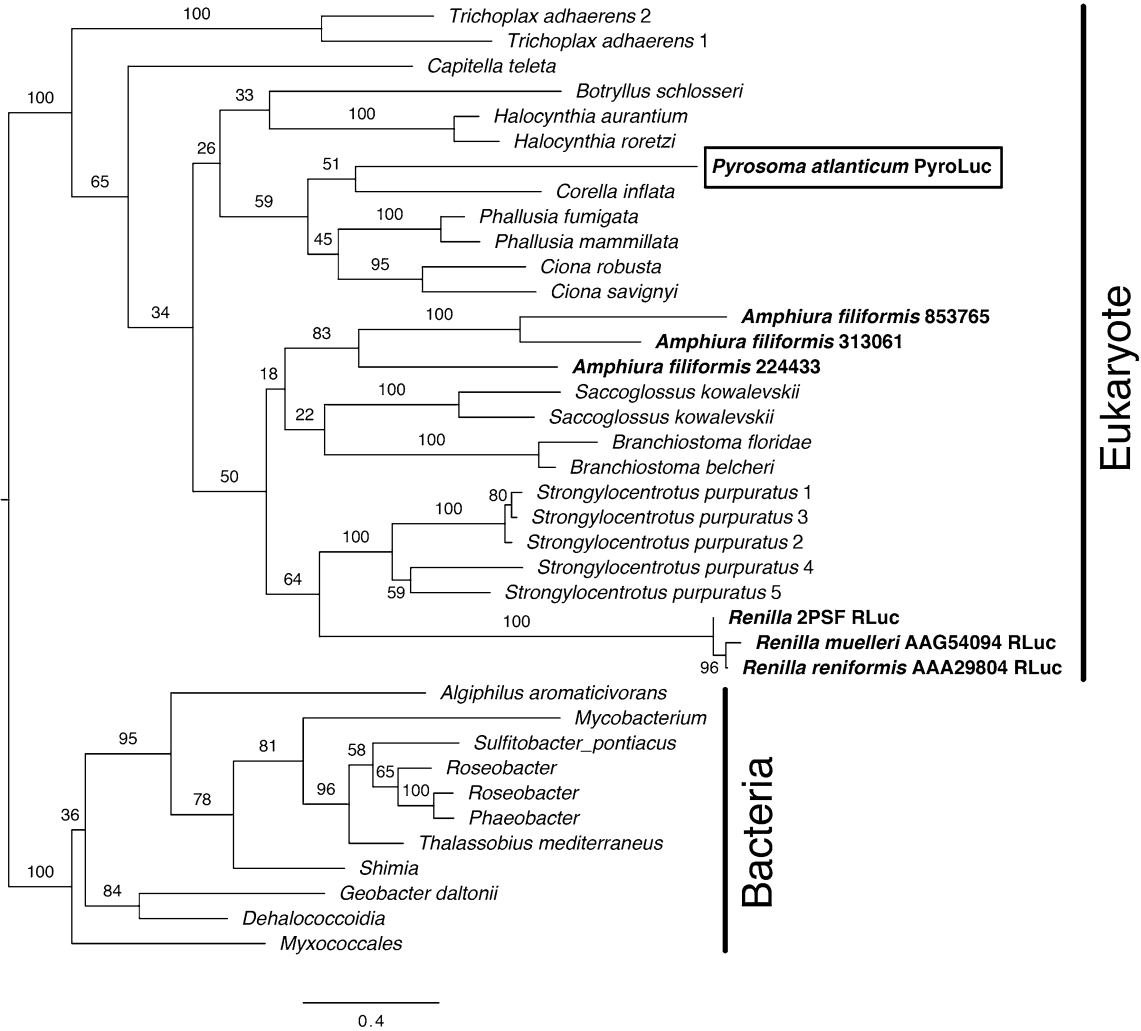
Maximum likelihood phylogeny of RLuc-like luciferases (bolded) and haloalkane dehalogenases. PyroLuc from *Pyrosoma atlanticum* is accentuated with a box. Support values are summaries of 1000 bootstrap replicates. The luciferases from *A. filiformis* have yet to be functionally confirmed, but are highly probable^29^.

### RACE PCR

RACE PCR on a Canadian *P. atlanticum* sample recovered the majority of the PyroLuc sequence with 100% identity, confirming the presence of PyroLuc in a second sample.

### Luciferase phylogenetics

A maximum likelihood phylogenetic reconstruction (Fig. 4) resulted in high support (100% bootstrap) for a eukaryote clade being seperated from a bacterial clade. Within the eukaryotic clade, the luciferases were not phylogenetically sister to one another. The pyrosome luciferase (PyroLuc) sequence was found to be within the eukaryote clade. More specifically, it was phylogenetically sister to *Corella inflata* (a tunicate), albeit with low support in maximum likelihood (51% bootstrap). All tunicates formed a clade.

### Immunolocalization of a *Renilla-*like luciferase protein

**Figure 5:**
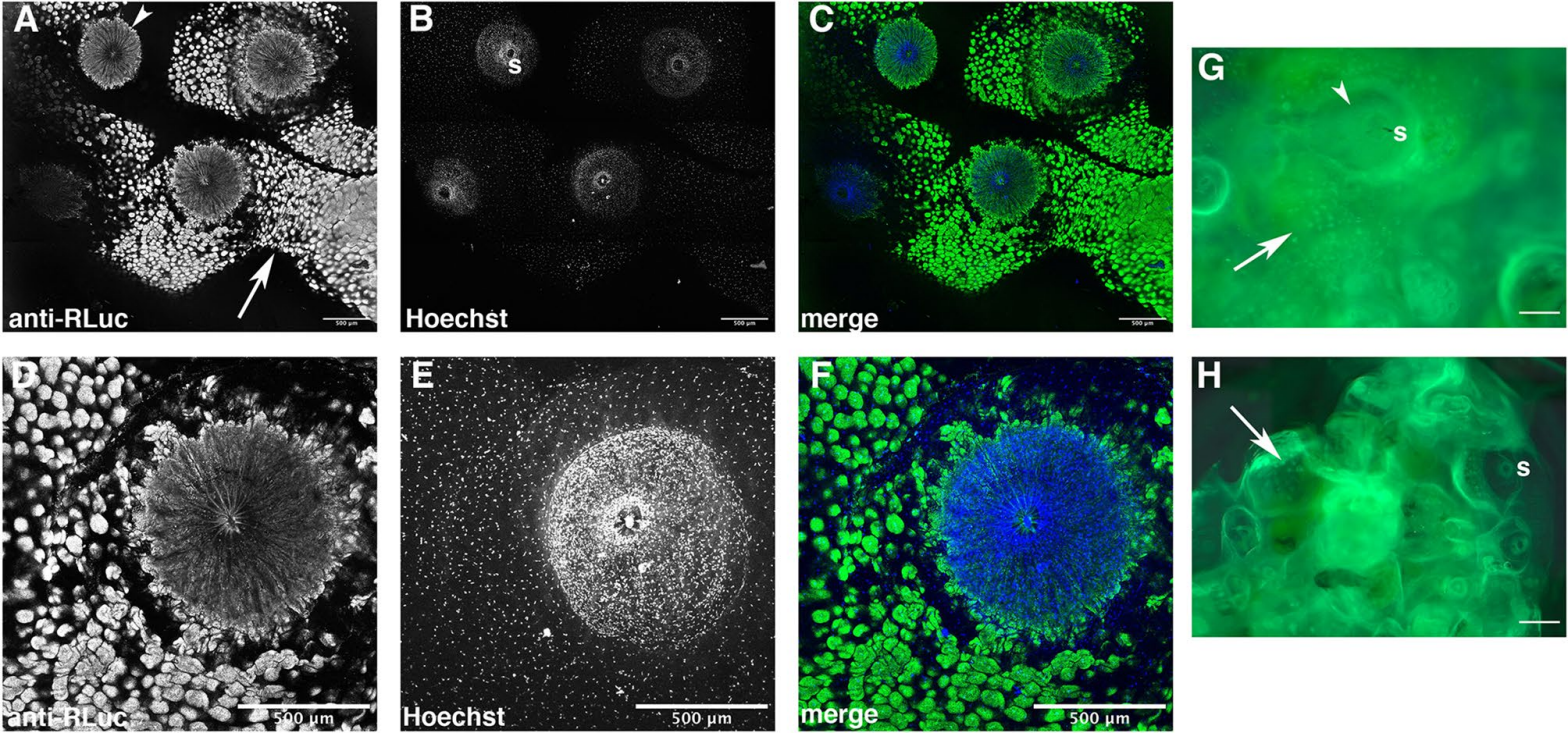
Expression of *Renilla*-like luciferase protein in *Pyrosoma atlanticum*. (**A**–**F**) Extended focus confocal projections of pyrosomes immunostained with an antibody to *Renilla* luciferase (anti-RLuc, green) and Hoechst (blue) to label nuclei. External views show incurrent siphons (s) of multiple (**A**–**C**) or single (**D**–**F**) zooids. Greyscale shown for A, B, D, and E; fluorescence shown for C and F. RLuc-like protein immunolocalizes to a large, circular structure underlying the incurrent siphon (**A**, arrowhead). (**G**–**H**) Fluorescent stereomicroscope images of a sample incubated with RLuc antibody (**G**) or rabbit pre-immune serum as a control (**H**). Individual patches of staining outside the siphon (arrows) appear to be localized to the tunic, and were shown to be non-specific using pre-immune serum (**G**, **H** and data not shown). Internal circular staining is specific to the RLuc antibody (**G**, arrowhead). Scale bars, 500 μm.

To attempt to detect a *Renilla-*like luciferase protein in pyrosome tissue, whole mount samples were fixed in 4% paraformaldehyde, treated with 1% Triton in PBS to permeabilize tissues, and incubated with an antibody that recognizes *Renilla* luciferase. This antibody was previously used to detect a *Renilla*-like luciferase in a brittle star^29^. Compared to samples incubated with pre-immune serum as a control, a strong signal was detected in each zooid in a circular area underlying the incurrent siphon, which is an average of 511 µm in diameter (n = 6) (Fig. 5A,C–D,F). The location and size of this circular structure is in the region of the luminous organ in samples examined by Mackie and Bone^10^. Tissue that was positive for RLuc was nucleated (staining using Hoechst stain), as would be predicted for a eukaryotic luciferase (Fig. 5B–C,E–F), but not a bacterial luciferase. Non-specific staining was detected as small, circular patches on the tunic (Fig. 5A,G–H) and low-level autofluorescence was observed (data not shown). While we have not generated a pyrosome-specific antibody, this data suggests that an RLuc-like protein is present in pyrosome tissues.

## Discussion

There are dozens of known bioluminescent systems, consisting of nine known natural luciferins, as well as dozens of complementary luciferases and photoproteins that have independently evolved^8,34,35^. Based on transcriptomics, phylogenetics, protein expression, and immunohistochemistry data, we present the first luciferase sequence putatively used by a chordate (*P. atlanticum*). This luciferase (PyroLuc) appears to be highly convergent with luciferases from two other phyla: Cnidaria (RLuc) and Echinodermata. Furthermore, like RLuc, PyroLuc reacts with coelenterazine. The first luciferase isolated and found to interact with coelenterazine was from a deep-water shrimp (*Oplophorus gracilirostris*)^36^. *Renilla* luciferase (RLuc) was among the first luciferases to be cloned^37^, and is the closest described enzyme to PyroLuc. RLuc is popular for bioimaging and other bioluminescent studies, as it can be expressed in numerous cell types. The properties and applications of PyroLuc in an experimental biology context are yet to be determined.

### Pyrosome bioluminescence

Bioluminescence is extremely common among marine animals. The most thorough quantitative study found that 76% of organisms in an area from shallow environments to the deep-sea emitted light^38^. This was similarly the case in the phylum Chordata: within tunicates, appendicularians were estimated to have 94% bioluminescent individuals; within vertebrates, fishes may have around 70% bioluminescent species^38^. However, putative molecular machinery behind luciferase production had not previously been indicated for any chordate. Given that our experiments mixing coelenterazine with *P. atlanticum* homogenate and this newly discovered PyroLuc both produced light, it appears probable that *P. atlanticum* uses coelenterazine as a luciferin. Coelenterazine is an imidazolopyrazinone luciferin found in bioluminescent organisms from at least nine phyla (from protozoans to vertebrates; not bacteria), and is of great importance in the evolutionary history of bioluminescence across the tree of life^8,29,39^.

Coelenterazine is found in many non-bioluminescent organisms, possibly obtained via diet^40^, and has strong antioxidative properties^41^. Most organisms that use coelenterazine as a luciferin do not synthesize it themselves, but they do typically produce their luciferases endogenously^8^. *Pyrosoma atlanticum*’s putative use of coelenterazine is congruent with the widespread use of this luciferin. Larvacean tunicates have also been shown to use coelenterazine^8^.

### The debate on bacterial bioluminescence in pyrosomes

It was initially proposed that pyrosome bioluminescence is due to bacterial symbionts, as the intracellular sources of light (“luminous cell”) resemble bacteria^42^. This hypothesis was furthered due to electron microscopy observations^10^ and by some associated bacterial luciferase activity^43^. However, our data provides some evidence supporting an endogenous coelenterazine-based luciferase as the source of bioluminescence in pyrosomes (bacteria do not use coelenterazine). Furthermore, our immunohistochemistry work highlights nucleated cells, which would not be present in bacteria. Still, these data are not sufficient to determine that bacteria are not used for pyrosome bioluminescence. Some other studies have rejected the hypothesis of symbiotic bacteria as the source of pyrosome luminescence due to consistently failed efforts to cultivate bacteria from luminous cells of *Pyrosoma*, as well as it being difficult to explain the wave-like mechanism of bioluminescence spreading across the colony^34^. Bioluminescence in bacteria is controlled via the lux operon and all other known examples of bacterial bioluminescence (flashlight fish, bobtail squid, etc.) exhibit non-modulatable bioluminescence, unlike that of *P. atlanticum* (Supp. Video 1).

### Convergent bioluminescence evolution across phyla

One of the most interesting points of discovery in this work is that a third phylum (Chordata) has at least one member (*P. atlanticum*) that putatively evolved a *Renilla*-like luciferase from the more widespread haloalkane dehalogenase gene family. A similar luciferase was recently predicted (yet to be expressed) from the brittle star *Amphiura filiformis*^29^. Based on our findings and others’^29^, the luciferases from these three phyla (Cnidaria, Echinodermata, and Chordata) appear to use coelenterazine as the luciferin.

The authors of the *A. filiformis* work elegantly connected the dots between the cnidarian and echinoderm luciferases with bacterial haloalkane dehalogenases, noting, for instance, a conserved amino acid triad in the luciferases^29^. Within animal RLuc-like genes, there also appears to be a conserved cysteine site that indeed is important for RLuc activity^29,33^. These other RLuc-like genes are from non-bioluminescent animal species, but they do share a notable level (> 44%) of similarity. While the function is not known for all of these species, at least one (DspA) to date has been confirmed to act as a haloalkane dehalogenase^44^. A few vertebrate sequences in GenBank are annotated as putative matches to haloalkane dehalogenase, but we are unaware of further research assessing the presence of these proteins in vertebrates.

Luciferases are oxygenases, and it is interesting that a light producing oxygenase could be derived multiple times from an enzyme with a rather different function (i.e., dehalogenases). However, this seems to be the case. Our phylogenetic results build on past work^29^ and indicate that, within animal RLuc-like genes, luminescence has evolved independently. PyroLuc is phylogenetically sister to a sequence from *C. inflata* and more generally nested within a clade of tunicate sequences. This helps show that PyroLuc is most likely of tunicate origin. It also indicates that this luciferase’s function evolved independently from the other RLuc-like genes, as the other tunicates examined are non-bioluminescent. (Although it is possible that this gene also has a bioluminescent capacity even if it is not used for this purpose in one or more of the other examined tunicates.) Furthermore, not only do these systems rely on coelenterazine, but their light emissions have roughly similar wavelengths: the blue-green light from *P. atlanticum* ranges from 475 to 493 nm^22–24^, while the brittle star light peaks around 472 nm and RLuc peaks at 480 nm^37^. Sequence variation in these convergent luciferases presumably cause these wavelength differences. Recent work has found that a single amino acid change next to the catalytic site of RLuc can make RLuc have both bioluminescence and dehalogenase functioning^45^. It would similarly not be surprising if one or a few amino acids could shift an ancestral dehalogenase to have luciferase functionality. However, we do not find any sites that converge between bioluminescent RLuc-like sequences in our study that are not found elsewhere, so we do not currently propose a specific site for future mutational work.

Other studies have found similar types of convergence from a single common gene source. Even within luciferases, the firefly luciferases are similar to those found in a sponge and a squid, and likely emerged from the widespread acyl-CoA ligases^29,46^.

### Areas of further investigation

It is worth noting that even the most comprehensive studies to date have later determined that transcripts were from potential prey items^47^. Along these lines, while transcriptomics and RACE PCR showed PyroLuc in two samples, it was not found in all transcriptomes. Since transcriptome analysis is dependent on the genes that are being expressed at a given time-point, it is possible that some samples were not producing the bioluminescent gene at the time of collection; however, it is hard to be sure about this. Furthermore, whereas our results indicate the luciferase system in *P. atlanticum* is likely coelenterazine-based, there is always the possibility that some other symbiont, such as bacteria, is at play in pyrosome luciferase production. In addition, immunohistochemistry in this study was performed using an Anti-RLuc antibody. While this antibody appears to be cross-reactive with PyroLuc, a specific anti-PyroLuc antibody does not yet exist, but would be useful.

Furthermore, it is still possible that many dehalogenases across non-bioluminescent animals may be capable of light production when exposed to coelenterazine, but are surely not using this luciferin. Additional work should be conducted into whether dehalogenases from non-bioluminescent animals produce light when exposed to coelenterazine. If these animals produce light, despite not being exposed to this luciferin in their environment, it would explain how easily this protein could be co-opted for light production if an animal consumes another organism that produces coelenterazine. However, it might also suggest that PyroLuc may not be used by the organism for light production.

It would be useful to identify the exact wavelength of PyroLuc luminescence using microspectrophotometry. In addition, mass spectrometry could be used to identify coelenterazine in the homogenate of the pyrosome. Lastly, designing a specific antibody for PyroLuc would be useful for further microscopy studies as well as analysis of the crude tissue by Western Blot.

## Conclusion

We believe this work adds important information regarding chordate bioluminescence for at least one species: the pyrosome *P. atlanticum*. Evidence for our conclusion—that this species likely uses a coelenterazine-based luciferase, similar to RLuc from a cnidarian—comes from transcriptomics, phylogenetics, coelenterazine experiments, expression data, and immunohistochemistry. We describe a novel luciferase that might be of utility in the growing molecular biology toolkit, given the usefulness of other structurally and functionally similar cnidarian RLuc luciferases.

## Methods

### Specimen collection

Seven specimens of *P. atlanticum* were collected on May 2017 in SE Brazil, near Alcatrazes Archipelago, using an Isaacs-Kidd Midwater Trawl as well as a Triton 3300/3 submarine with a soft robotic arm operated via a haptic glove^11^ (Fig. 1E and Supp. Video 4); soft robotics appear not only to reduce physical damage, but also to cause less stress-induced transcriptional changes^48^. These specimens were collected under Permit # Sisbio 57721 from the Instituto Chico Mendes de Conservação da Biodiversidade (ICMBio), Brazilian Ministry of the Environment.

Hundreds of *P. atlanticum* specimens were collected using the CCGS John P Tully between July 21–26, 2017 off Vancouver Island, Canada (Supp. Figure 2), as part of the ongoing Line P Monitoring and La Perouse Zooplankton Monitoring programs run by Institute of Ocean Sciences (IOS)—Ocean Science Division^12,13^. Bongo nets were deployed off the aft deck, lowered at a rate of 0.5 m/s and retrieved at 1 m/s. The bongo net consists of two black cylindrical–conical nets mounted on a central towing frame and weight. Each net has a 0.25 m^2^ mouth area, a filtering area/mouth area ratio of 11.5, and 0.23 mm aperture black mesh. Volume filtered is measured by a TSK flowmeter mounted in the mouth of one net. Tow depths (determined from wire out and wire angle) followed established time series protocols for the offshore and continental margin regions: near-bottom-to-surface or 250 m-to-surface. Time from the net to the − 80 °C freezer was kept to less than 10 min for all pyrosomes. The *P. altlanticum* specimens used in this study represent a disparate geographic range (Supp. Figure 2).

### Transcriptomic sequencing and analysis

An RNeasy Fibrous Tissue Mini Kit (Qiagen #74704) was used to extract RNA from these *P. atlanticum* samples. The two highest quality extractions from Brazil and the two highest quality extractions from Canada were then used for transcriptomic sequencing at the New York Genome Center using a HiSeq 25000 (125 × 125 bp). Sequences are in the Short Read Archive under BioProject PRJNA667300.

Sequences were processed following our prior work^49,50^. In short, assemblies were produced using Trinity 2.4 with sequences first being trimmed with Trimmomatic^51^. Transdecoder 3.0^52^ was then used to call open reading frames (ORFs); a 5 amino acid minimum was established to allow for searches of possibly short luciferins. We used ORF sequences as blastp queries against the local databases of luciferases and photoproteins from our prior work^50,53^. Queries using blastp against these local databases used an e-value minimum cutoff of e^−5^. Matches meeting this cutoff were then reciprocally used as blastp queries against Swissprot/Uniprot to confirm that no better match was found in a well-curated database. If bitscores were better or equal for our local blastp searches, the sequence was considered a putative match. Bitscores were used instead of e-values, as they do not rely on database size which is highly skewed between local searches and large databases. Any identified proteins of interest (e.g., luciferases) were modeled for homology with Swiss-Model^54^ using the default parameters.

### Novel luciferase expression and bioluminescence assays

PyroLuc was successfully synthesized and expressed in *E. coli* Origami DE3 (Novagen). The gene for PyroLuc was cloned into a pET-45b( +) vector with an N-terminal His tag. Cloning was done by Genscript U.S.A. A starter culture of Origami DE3 (Novagen) was grown at 37° C overnight. Larger cultures were inoculated with the starter culture and 100 mM IPTG was used for induction once cells reached an O.D. 600 of 0.6. Following induction, the culture was grown at 37° C for 3 h. Induction pellets were washed in 1% PBST buffer (1X PBS and 1% Triton X-100), pH 7.4, and centrifuged at 6000 rpm for 20 min. The supernatant was discarded and the pellet was then resuspended in 15 mL of 1% PBST buffer with 10 mM DTT, pH 7.4. The resuspension was sonicated at 100% amplitude for 10 min (30 s bursts with 1-min breaks), and the lysate was then centrifuged at 8000 rpm for 20 min. Supernatant was again discarded and the pellet was resuspended in 25 mL 1% PBST with 8 M Urea and 10 mM DTT, pH 7.4. The solubilized supernatant was then run through the column. We refolded the protein on the Nickel NTA column using a series of refolding buffers with decreasing urea concentration (8 M Urea, 6 M Urea, 4 M Urea, 2 M Urea, and 0 M Urea) in 1X PBS, pH 7.4. PBS with 300 mM imidazole, pH 7.4, was used to elute the protein. A gel of the protein purification is shown in Supp. Figure 5. The identity of PyroLuc was confirmed by mass spectrometry analysis (MS BioWorks, Ann Arbor MI) of a gel band following protein purification. Bioluminescent assays were conducted on a Spectra Max-L Microplate reader (Molecular Devices, San Jose CA) using an emission of wavelength of 480 nm, consistent with coelenterazine based luciferases.

### RACE PCR

RACE PCR was used to validate the presence of our PyroLuc in another Canadian sample. Specifically, we performed 3′ RACE System for Rapid Amplification of cDNA Ends (ThermoFisher# 18373-019) and 5′ RACE System for Rapid Amplification of cDNA Ends (ThermoFisher# 18374-058).

### Luciferase phylogenetics

The luciferase found in *P. atlanticum* was combined with a matrix of luciferases and haloalkane dehalogenases. We compiled these sequences using those from a study focussing on *A. filiformis* luciferase^29^. The sequences on ANISEED^55^ were taken by conducting a tblastn search against each tunicate genome that had gene models available, with a query of PyroLuc and the similar *Ciona robusta* sequence from the prior study^29^ (*C. intestinalis* in that study; please see the following paper regarding taxonomic changes in this lineage^56^). Putative matching sequences were then searched for reciprocally as blastp queries against Swissprot/Uniprot. Only hits that did not find better matches in this search or better matched a known dehalogenase or luciferase sequence were kept. In essence, we kept sequences that appeared to be dehalogenases or luciferases. The same was done for *Branchiostoma belcheri* from GenBank. The matrix was processed following our prior work^50^: alignments (Supp. Data 1) were produced via MUSCLE v3.8.31^57^, and then a phylogenetic reconstruction of the data was produced using 1000 bootstrap replicates for support with the LG + I + G4 model (picked using automatic model selection) in IQ-Tree multicore version 2.0.5^58^ in the CIPRES Science Gateway^59^. Bacterial haloalkane dehalogenases were used as outgroup taxa.

### Imaging of bioluminescence

Immediately following collection in Brazil, *P. atlanticum* was brought into a dark aquarium room and stimulated (either mechanically or photically with a Nikon Speedlight SB-910 strobe) to initiate bioluminescence. Videos and stills were taken on a Sony A7s II camera.

### Immunohistochemistry

Pyrosomes were fixed in 4% paraformaldehyde in PBS for 20 min and then incubated in 0.5% Triton in PBS for 1 h at room temperature on a rocking nutator. Samples were then blocked in PBT-BSA (0.5% Triton X-100, 0.5% bovine serum albumin) for 30 min at room temperature. An anti-*Renilla* luciferase antibody (1:250, GTX125851, GeneTex) or rabbit pre-immune serum (1:250, ab37415, Abcam) was added and samples were incubated at 4 °C overnight. GTX 125851 is a polyclonal antibody with specificity for *Renilla* luciferase. Samples were then washed in PBS and incubated with AlexaFluor-488-conjugated anti-Rabbit secondary (1:200, Invitrogen) for 1 h at room temperature. Following secondary antibody incubation, samples were washed in PBS and Hoechst 33342 (Molecular Probes, 1:2000) was added during a 10 min wash in PBS. Samples were dissected and placed in a glass-bottomed petri dish with PBS for imaging. In addition to the pre-immune serum control, other control samples were incubated without primary or secondary antibody, and no specific signal was observed (data not shown).

### Confocal imaging

Confocal images were acquired on a Zeiss 880 Airyscan Live Cell laser-scanning confocal microscope equipped with a 10 × 0.30 NA M27 EC Plan-Neofluor objective and ZEN Black software. Maximum intensity projections were rendered using Fiji (Image J) software. Fluorescent images were obtained on a Zeiss Pentafluor Discovery V8 stereomicroscope equipped with a 0.63X Achromat FWD 107 mm objective lens and ZEN Blue software. Images were processed using Adobe Photoshop CC.

## Supplementary information

Supplementary Information 1.

Supplementary Information 2.

Supplementary Information 3.

Supplementary Information 4.

Supplementary Information 5.

Supplementary Information 6.

Supplementary Information 7.

Supplementary Information 8.

Supplementary Information 9.

Supplementary Information 10.

Supplementary Information 11.
