# Supplementary figures and images for: A putative chordate luciferase from a cosmopolitan tunicate indicates convergent bioluminescence evolution across phyla
Source: Sci Rep. 2020 Oct 20;10:17724. doi: 10.1038/s41598-020-73446-w (PMC7576829; doi:10.1038/s41598-020-73446-w)

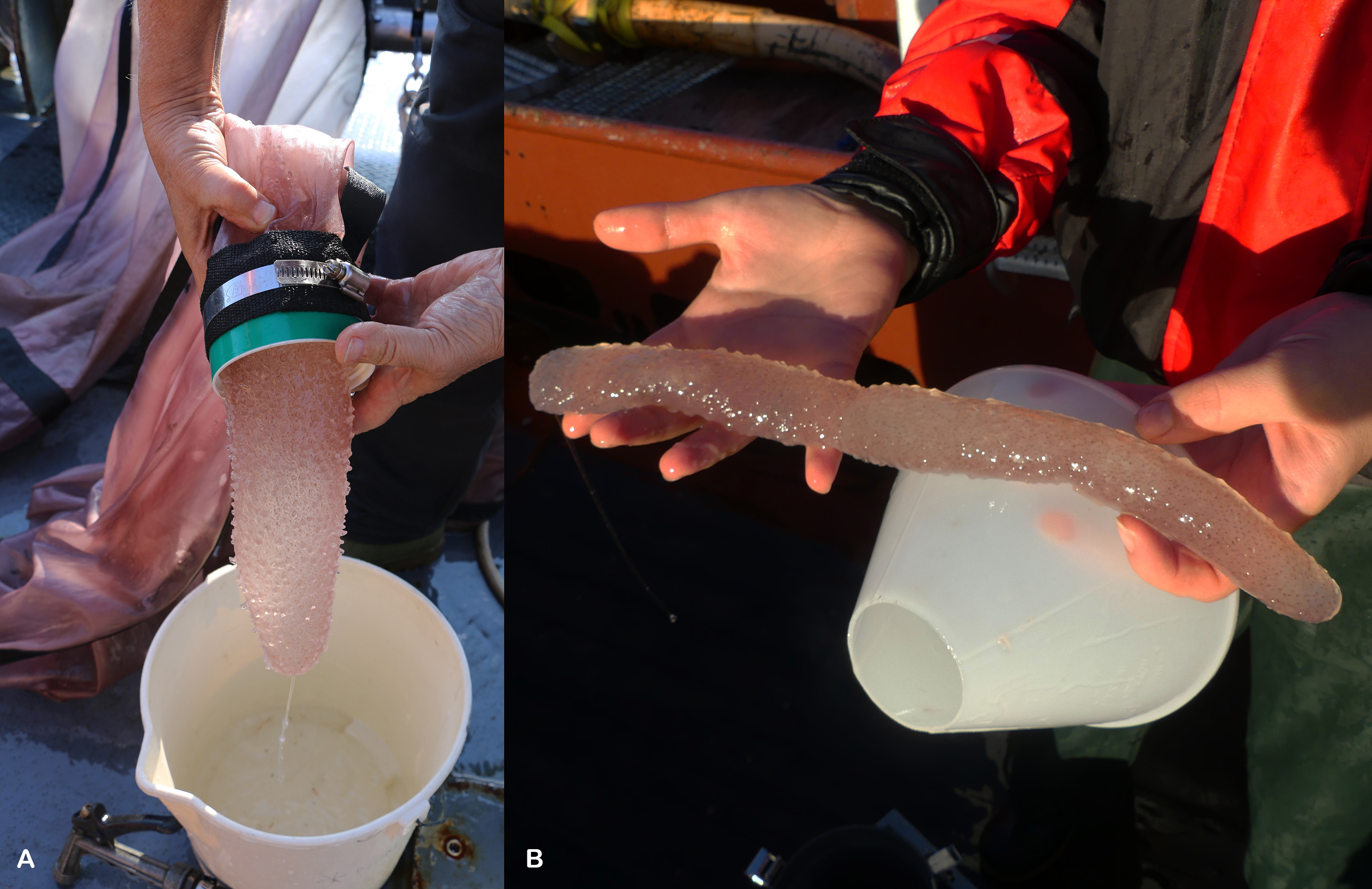

Supplement: Supplementary file 2 — Supplementary Information 2. [file 41598_2020_73446_MOESM2_ESM.jpg]

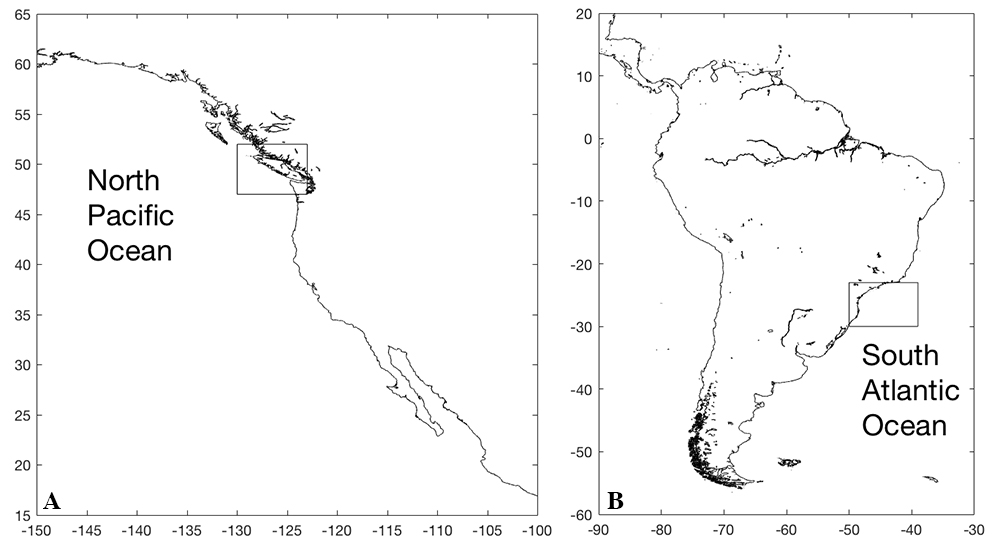

Supplement: Supplementary file 3 — Supplementary Information 3. [file 41598_2020_73446_MOESM3_ESM.jpg]

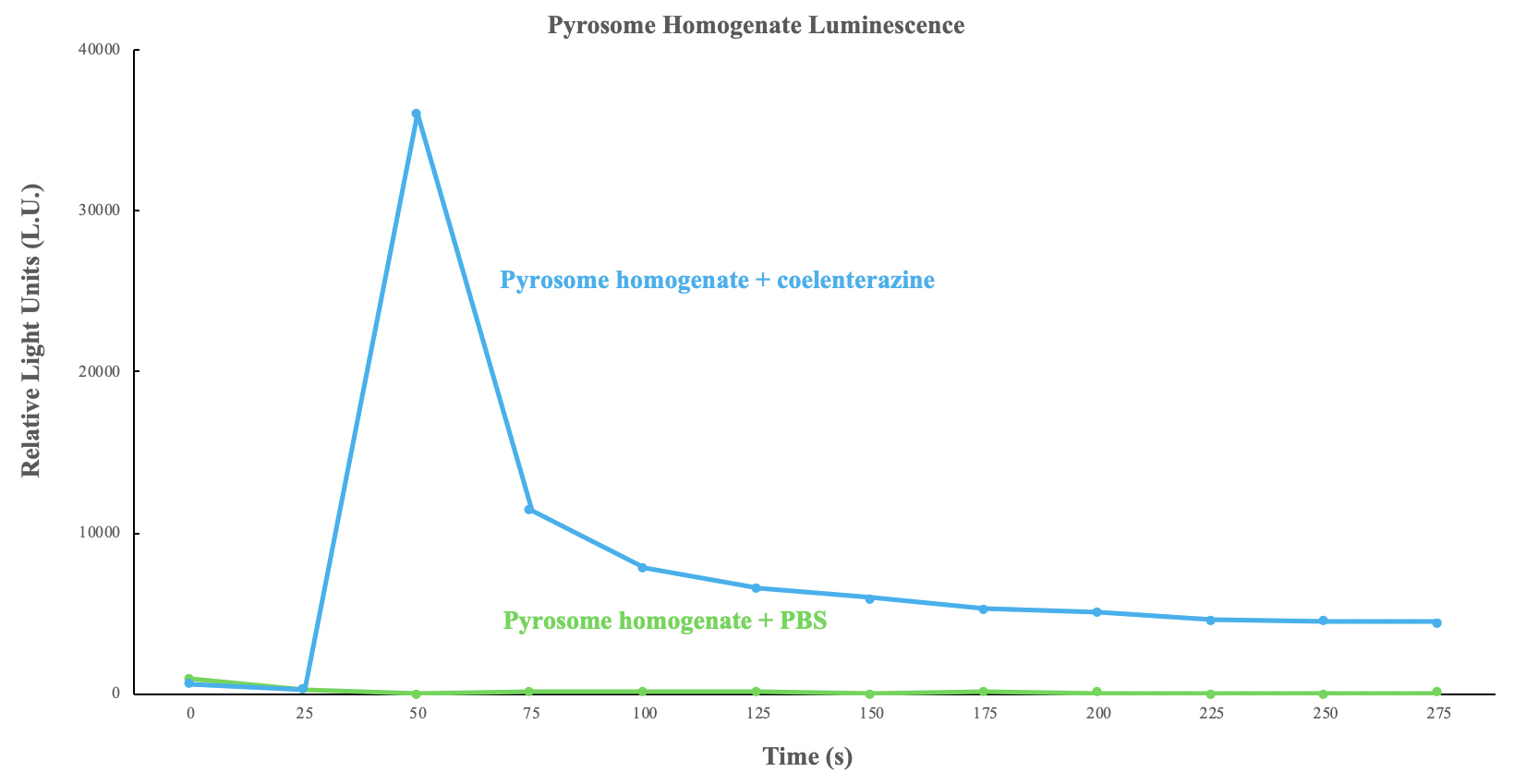

Supplement: Supplementary file 4 — Supplementary Information 4. [file 41598_2020_73446_MOESM4_ESM.jpg]

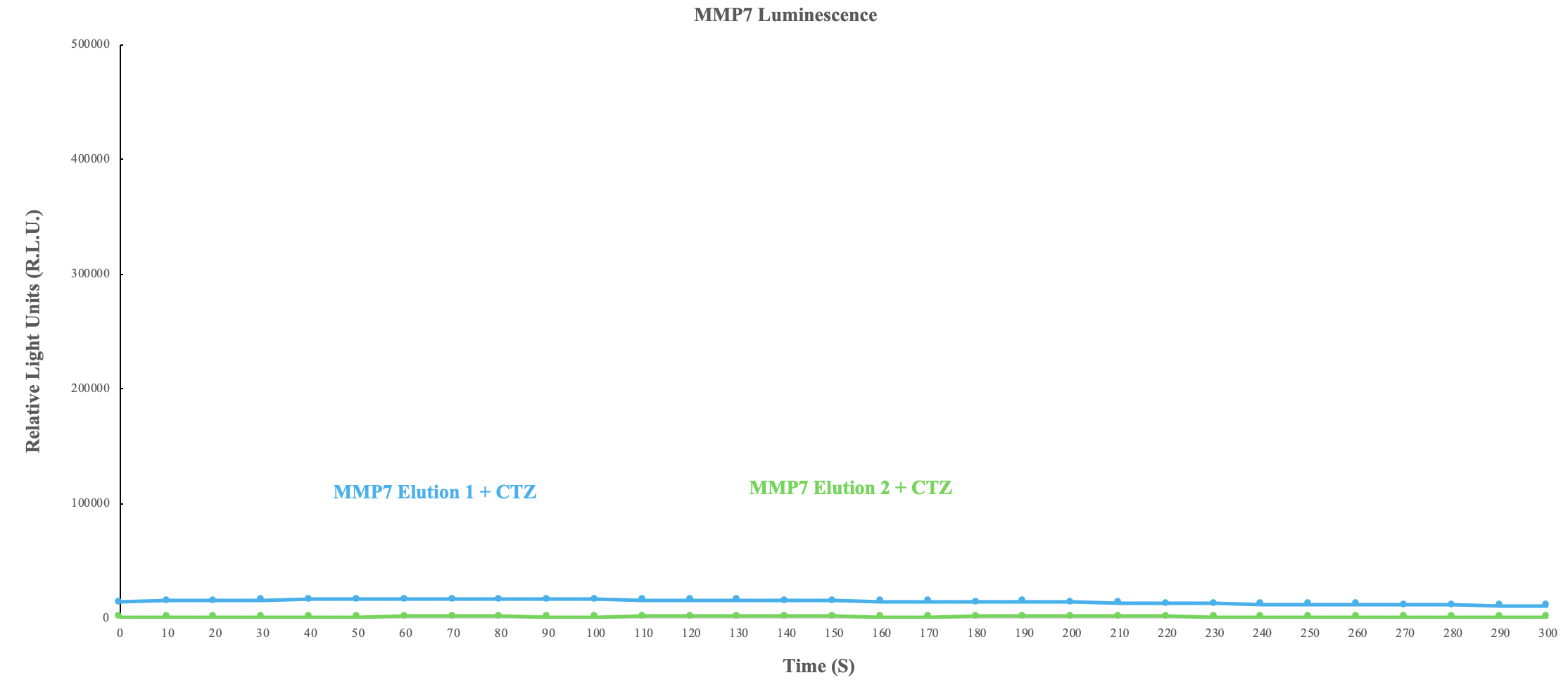

Supplement: Supplementary file 5 — Supplementary Information 5. [file 41598_2020_73446_MOESM5_ESM.jpg]

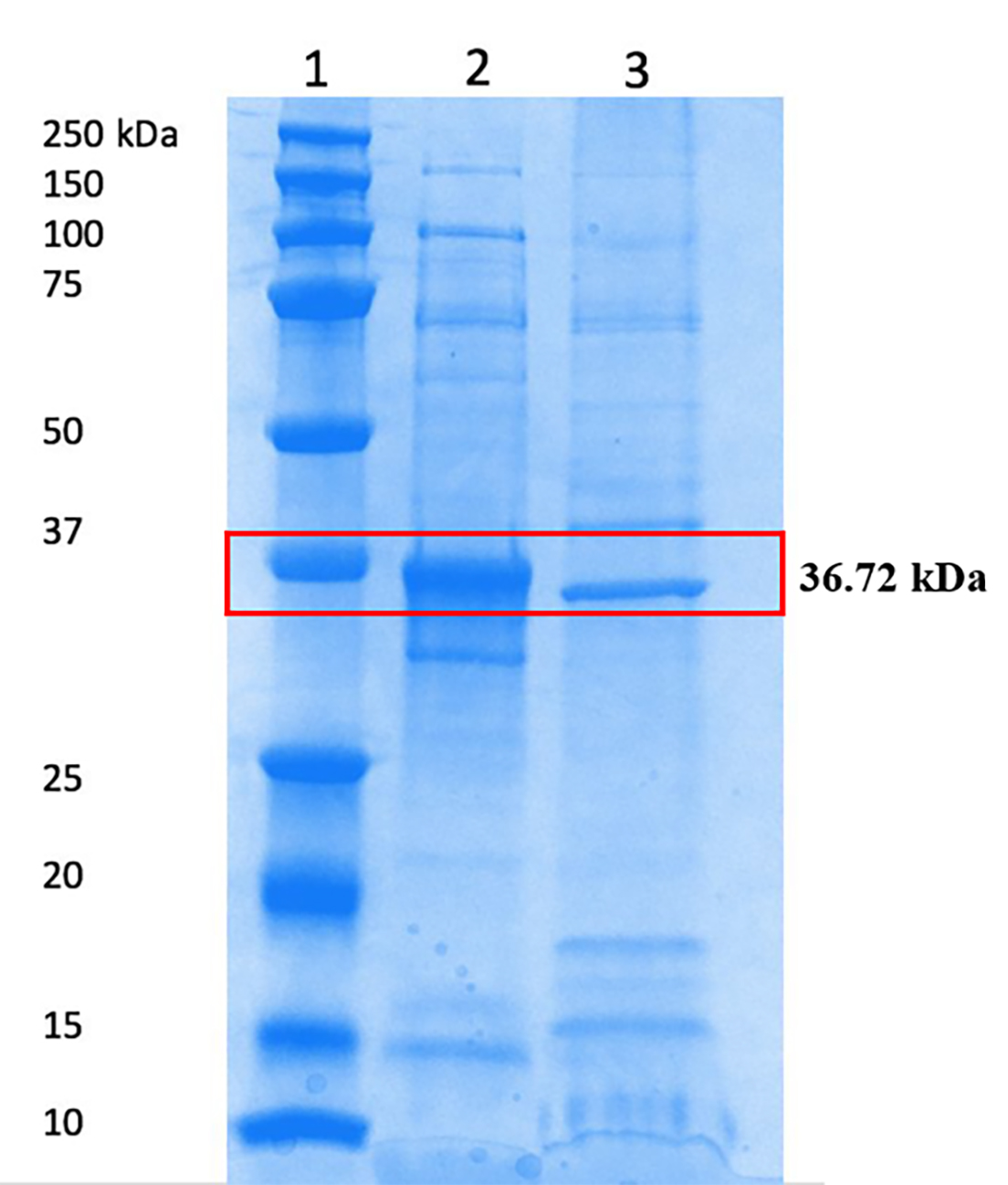

Supplement: Supplementary file 6 — Supplementary Information 6. [file 41598_2020_73446_MOESM6_ESM.jpg]
