# Supplementary material for: A putative chordate luciferase from a cosmopolitan tunicate indicates convergent bioluminescence evolution across phyla
Source: Sci Rep. 2020 Oct 20;10:17724. doi: 10.1038/s41598-020-73446-w (PMC7576829; doi:10.1038/s41598-020-73446-w)
Supplement: Supplementary file 11 — Supplementary Information 11. [file 41598_2020_73446_MOESM11_ESM.docx]

**A putative chordate luciferase from a cosmopolitan tunicate indicates convergent bioluminescence evolution across phyla**

Michael Tessler ^1, A, *^, Jean P. Gaffney ^2, 3, A, *^, Anderson G. Oliveira ^4^, Andrew Guarnaccia ^2, 3,^ , Krista C. Dobi ^2, 3^, Nehaben A. Gujarati ^2^, Moira Galbraith ^5^, Jeremy D. Mirza ^4, 6^, John S. Sparks ^1, 7^, Vincent A. Pieribone ^8^, Robert J. Wood ^9^, and David F. Gruber ^1, 2, 3, *^

^1^ Sackler Institute for Comparative Genomics, American Museum of Natural History, New York, NY 10024, USA. ^2^ Baruch College, City University of New York, Department of Natural Sciences, New York, NY 10010, USA. ^3^ The Graduate Center, PhD Program in Biology, City University of New York. ^4^ Departamento de Oceanografia Física, Química e Geológica, Instituto Oceanográfico, Universidade de São Paulo, 05508-120, Brazil. ^5^ Institute of Ocean Sciences, 9860 West Saanich Road, P.O. Box 6000, Sidney, B.C. V8L 4B2. ^6^ Departamento de Química, Instituto de Ciências Ambientais, Químicas e Farmacêuticas, Universidade Federal de São Paulo, Diadema, São Paulo, Brazil. ^7^ Department of Ichthyology, Division of Vertebrate Zoology, American Museum of Natural History, New York, NY 10024, USA. ^8^ Cellular and Molecular Physiology, Yale University, New Haven, CT, United States of America. ^9^ Wyss Institute for Biologically Inspired Engineering, Harvard University, Cambridge, MA, USA.

^A^ Authors contributed equally.

^*^ Correspondence: mtessler@amnh.org, jean.gaffney@baruch.cuny.edu, and david.gruber@baruch.cuny.edu

**Supplementary Information**

**Supp. Video 1.** *Pyrosoma atlanticum* bioluminescence.

A compilation of manually stimulated *Pyrosoma atlanticum* bioluminescence taken <15 minutes after collection in Brazil. Video courtesy of OceanX.

**Supp. Video 2.** *Pyrosomella verticillata* bioluminescence.

Manual stimulation of *Pyrosomella verticillata* bioluminescence. Video courtesy of OceanX.

**Supp. Video 3.** *Pyrosoma atlanticum* bioluminescence decay kinetics.

Video of bioluminescence decay kinetics following light stimulated bioluminescence in *Pyrosoma atlanticum.*

**Supp. Video 4.** Biological collection using soft robotics.

Collection of P*yrosoma atlanticum* from the water column with a soft robotic arm on a Triton 3300/3 submarine. Brennan Phillips operating teleoperated device (left), David Gruber (right) and Mark Taylor, submarine pilot (center). Informed Consent to publish obtained. Video courtesy of OceanX.

**Supp. Data 1.** Sequences for alignments.

Alignments of *Renilla*-like luciferases and haloalkane dehalogenases.

**Supp. Fig. S1 A, B)** Collection of *Pyrosoma atlanticum* from the Canadian Pacific during a rare northern bloom using a Bongo plankton net. Colonies from this bloom reached over 80 cm in length.

**Supp. Fig. S2** Collection locations of *Pyrosoma atlanticum* used in this study: A) Canada and B) Brazil.

**Supp. Fig. S3** Pyrosome tissue homogenate and control in PBS buffer, pH 7.4.

**Supp. Fig. S4** Coelenterazine (24.5 µM) reaction with matrix metalloproteinase-7 (21.9 µM MMP7 Elution 1, 3.8 µM MMP7 Elution 2) as a negative control in PBS, pH 7.4.

**Supp. Fig. S5** PAGE gel of PyroLuc. Lane 1, BioRad molecular weight ladder. Lane 2, solubilized pellet, supernatant. Lane 3, Purified PyroLuc following elution from Ni-NTA resin. Protein identity was confirmed by mass spectrometry analysis (MS Bioworks, Ann Arbor MI) of a Coomassie stained gel band.
